# Hyperspectral IASI L1C Data Compression

**DOI:** 10.3390/s17061404

**Published:** 2017-06-16

**Authors:** Joaquín García-Sobrino, Joan Serra-Sagristà, Joan Bartrina-Rapesta

**Affiliations:** Department of Information and Communications Engineering, Universitat Autònoma de Barcelona, 08193 Bellaterra, Spain; joan.serra@uab.cat (J.S.-S.); joan.bartrina@uab.cat (J.B.-R.)

**Keywords:** IASI instrument, hyperspectral remote sensing, data compression, lossless, near-lossless and lossy compression

## Abstract

The Infrared Atmospheric Sounding Interferometer (IASI), implemented on the MetOp satellite series, represents a significant step forward in atmospheric forecast and weather understanding. The instrument provides infrared soundings of unprecedented accuracy and spectral resolution to derive humidity and atmospheric temperature profiles, as well as some of the chemical components playing a key role in climate monitoring. IASI collects rich spectral information, which results in large amounts of data (about 16 Gigabytes per day). Efficient compression techniques are requested for both transmission and storage of such huge data. This study reviews the performance of several state of the art coding standards and techniques for IASI L1C data compression. Discussion embraces lossless, near-lossless and lossy compression. Several spectral transforms, essential to achieve improved coding performance due to the high spectral redundancy inherent to IASI products, are also discussed. Illustrative results are reported for a set of 96 IASI L1C orbits acquired over a full year (4 orbits per month for each IASI-A and IASI-B from July 2013 to June 2014) . Further, this survey provides organized data and facts to assist future research and the atmospheric scientific community.

## 1. Introduction

The Infrared Atmospheric Sounding Interferometer (IASI) is a new generation of nadir viewing instruments for obtaining atmospheric measurements with unprecedented quality. The acquired data provides useful information for many application areas such as meteorology, climate monitoring or atmospheric chemistry. IASI data is recorded with high spectral accuracy, producing more than 8000 spectral channels that need be stored and transmitted.

The literature on IASI data is extensive. IASI products provide qualitative data for a wealth of possibilities such as numerical weather prediction (NWP) [1]; for studying the essential climate variables as cloud properties, greenhouse gases, or the hydrological cycle evaluation [2]; for predicting temperature and water vapor profiles [3,4]; or for analyzing several chemical atmospheric components (CO, CO${}_{2}$, CH${}_{4}$, SO${}_{2}$, N${}_{2}$O, HNO${}_{3}$, NH${}_{3}$, OCS, and CF${}_{4}$) [5,6,7,8,9,10,11]. The high resolution of the data also allows to examine the composition of the lowest part of the atmosphere, enabling the research of specific events. For instance, Coheur et al. [12] and Turquety et al. [13] use IASI data to study the chemical composition deep in the troposphere to track the emission and movement of pollution from wildfires.

The high definition of the sensor in terms of spectral, spatial, and temporal resolution produce collected data with a considerably large size: about 16 Gigabytes per day in Binary Universal Form (BUFR) for the Representation of meteorological data format. IASI covers the spectral range between 645 and 2760 cm${}^{-1}$. In each acquisition, 8359 spectral channels are acquired on the satellite, the IASI processing chain [14] leads to 8461 channels distributed on Earth, thus yielding a large volume of information, which is costly to manage in an operational context, i.e., for transmission and storage. An effective way to alleviate the large amount of data produced by the instrument is to compress the IASI products according to the specific needs of the final users.

In the IASI community, Principal Component Compression (PCC) is an accepted approach for compression of IASI data. PCC is a lossy compression strategy intended to produce a truncated Principal Components (PC) representation; additionally, it allows to reduce the dimensionality of the data [15,16,17,18,19,20]. Although PCC is a mature field in the scope of IASI dimensionality-reduction and of IASI compression, other data compression techniques can also produce competitive performance for compression of IASI spectra.

In the framework of remote sensing data compression, three data coding paradigms can be adopted: lossless, near-lossless, or lossy compression. Lossless compression allows perfect reconstruction but achieves low compression ratios. Lossy compression introduces distortion in the reconstructed data while achieving high compression ratios. Near-lossless compression introduces a restricted distortion and achieves moderate compression ratios [21,22].

In some remote sensing applications, lossy compression techniques are still appropriate because several application-oriented processes do not reduce their performance even for large levels of distortion [23,24,25,26,27]. Furthermore, lossy compression is acknowledged in new remote sensing missions because the inherent data acquisition noise is usually larger than the distortion introduced during the coding process [28].

Near-lossless paradigm is a particular kind of lossy compression. The data quality is controlled by selecting a maximum acceptable distortion error, usually the Peak Absolute Error (PAE), between each sample of the original and the reconstructed data. Near-lossless coding is convenient when efficient data transmission or storage is intended and preserving a specific accuracy of the recovered data is requested.

This paper puts forward a comprehensive review of the compression performance of several lossless, near-lossless, and lossy coding techniques for IASI L1C products, which are the most common format for distribution of IASI data. In particular, the following six recent coding techniques and standards will be considered and their performance assessed: JPEG-LS [29], JPEG 2000 [30], M-CALIC [31], CCSDS-122.0 [32], CCSDS-123.0 [33] and HEVC [34]. Also, four spectral transform will be paired along with these coding techniques to exploit the high spectral redundancy inherent to IASI data (over 8000 channels); specifically, we will look upon Karhunen-Loève Transform (KLT) [35], Wavelet Transform (WT) [36], Pairwise Orthogonal Transform (POT) [37], and Regression Wavelet Analysis Transform (RWA) [38].

To provide a quantitative and qualitative comparison and an accurate analysis, a representative set of 96 IASI L1C products has been thoroughly selected over a full year, from July 2013 to June 2014. The same number of orbits have been selected from each instrument, 48 orbits from MetOp-A and 48 orbits from MetOp-B, one orbit per week. The selection criteria have considered different areas, seasons, and acquisition time.

This investigation reviews effective strategies and furnishes instructions and recommendations to improve the transmission and storage of IASI L1C products, which can benefit the development of prevailing and upcoming high spectral resolution infrared instruments.

The remaining part of the paper is organized as follows. Section 2 briefly introduces the space program operating IASI, details of the instrument, and the processing performed since the data are acquired by the instrument until they are disseminated to end-users. Section 3 introduces the basic scheme of a data coding system, the characteristics of the coding techniques assessed, puts forward the setting and parameters of each technique, and states the benefits of applying a spectral transform along the spectral dimension. Section 4 reports the experimental results and provides analysis and an extensive discussion. Finally, Section 5 draws some conclusions.

## 2. IASI Instrument

This section reviews the operational structure of the IASI instrument. First, the basic structure of the space program is outlined. Then, details of the architecture and the operating mode of the instrument are described. Finally, the main stages of the processing chain are summarized, explaining how interferograms captured by the sensor are transformed into end-user products and disseminated to data centers.

### 2.1. Space Program of IASI Instrument

IASI instrument is implemented on the MetOp satellite series, which is part of the European Organization for the Exploitation of Meteorological Satellites (EUMETSAT) Polar System (EPS). The mission is led by EUMETSAT in cooperation with American scientific agency National Oceanic and Atmospheric Administration (NOAA). Both organizations hold close collaboration through the Initial Joint Polar System (IJPS). The MetOp satellites carry a set of instruments provided by NOAA and a new generation of European instruments, IASI among them. The main objective of the program is to harvest and exchange environmental data between EUMETSAT and NOAA and disseminate the collected information to the scientific community in support of global climate monitoring and NWP [39,40], where IASI represents the principal instrument of the mission.

The EPS comprises both space and ground components. The space component consists of the MetOp satellite series (MetOp-A, MetOp-B, and MetOp-C), which are being jointly developed by EUMETSAT and the European Space Agency (ESA). While MetOp-A and MetOp-B were launched in October 2006 and September 2012, respectively, MetOp-C is planed to be launched in October 2018 [41]. The recent extension of MepOp-A useful lifetime to 2022 [42] will enable joint operation of MetOp-A, MetOp-B, and MetOp-C from 2019 onwards. The operation of the three IASI instruments on-board of the MetOp satellite series will provide over 20 years of continuous observations, which represents a statistically significant series of climate variables.

**Figure 1:**
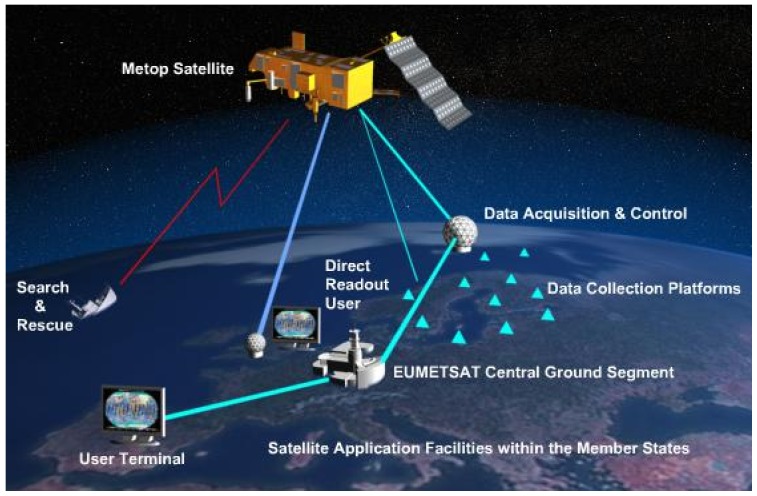
EPS program elements. The space component comprises the MetOp-A, MetOp-B, and MetOp-C satellites, while the ground component includes reception and operating stations.

The ground component of the program consists of several reception and operating stations responsible for collecting, operating, processing and distributing the collected data. Figure 1 (courtesy of EUMETSAT) illustrates the operational mode of the EPS program. Specific and more detailed elements of the program can be found in [43,44].

### 2.2. IASI Instrument Details

IASI instrument is the result of a cooperating agreement between EUMETSAT and the French Centre National d’Études Spatiales (CNES). CNES is responsible for the IASI instrument development and the data processing software, while EUMETSAT has the responsibility of storage, distribution, and exploitation of IASI data.

**Figure 2:**
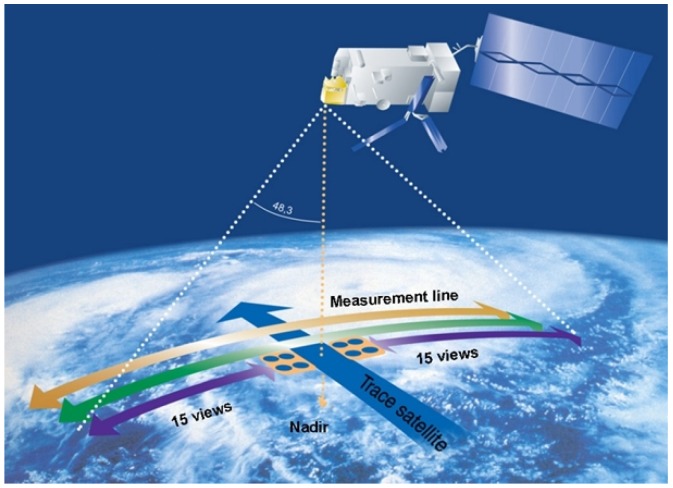
Modus operandi of IASI instrument. The instrument scans the Earth’s surface at regular intervals producing 30 FORs per line. Each FOR consists of 4 IFOVs, each of which represents a full spectrum.

**Figure 3:**
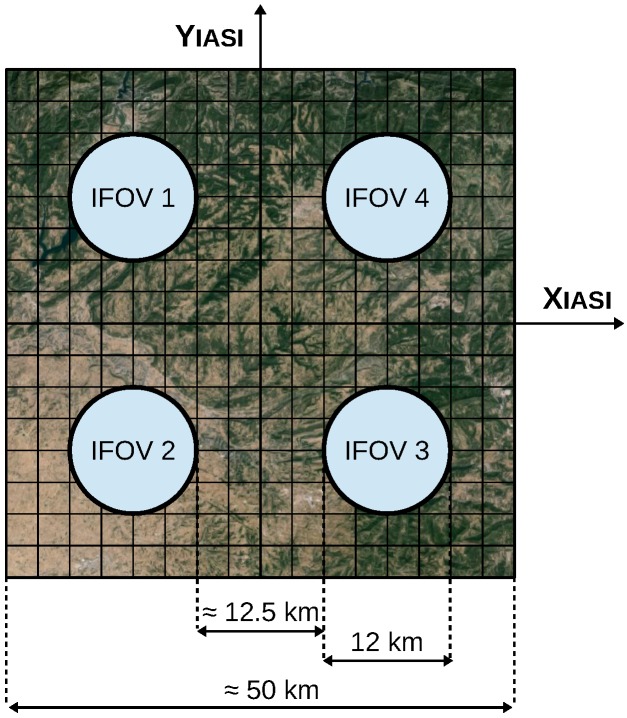
FOR and IFOV details. A single FOR consists of 4 IFOVs. Each IFOV spreads 12 km of the Earth’s surface and is separated from its neighboring IFOVs by 12.5 km. Each FOR corresponds to, approximately, 50 km of the Earth’s surface.

The instrument collects data over a horizontal swath width of, approximately, 2200 km. This ensures 99% global coverage of the Earth’s surface performed every 12 h (2 times per day), which means 14 daily orbits in a sun-synchronous mid-morning orbit [39]. IASI observes the Earth’s surface in a step and stare mode with fast movements between different look locations and stop during the acquisition of interferograms (see Table 1). The scanning process gathers atmospheric soundings on both sides of the vertical line along 30 look positions spaced by approximately 3.3 degrees. The optical axis moves from −47.85 degrees to +47.85 degrees with respect to the nadir position [45]. The scanning process takes 8 seconds per line and produces 30 elementary fields of regard (FOR) that correspond to 30 mirror positions. Each FOR consists of a 2×2 matrix of instantaneous fields of view (IFOV), matching to four circular pixels of the captured orbit. Each IFOV represents a full spectrum that is acquired in 3 bands: 645–1240 cm${}^{-1}$, 1200–2040 cm${}^{-1}$, and 1960–2760 cm${}^{-1}$ [14,46]. Each collected IFOV spreads 12 km of the Earth’s surface and is separated from another IFOV by 12.5 km, so that each FOR covers, approximately, 50 km at nadir position. Figure 2 (courtesy of EUMETSAT) illustrates the modus operandi of the instrument. Figure 3 displays FOR characteristics and IFOV numbering [46,47]. Table 1 summarizes the main characteristics of IASI instrument. Further IASI technical descriptions can be found in [3,45,48,49].

### 2.3. IASI Processing Chain

**Figure 4:**
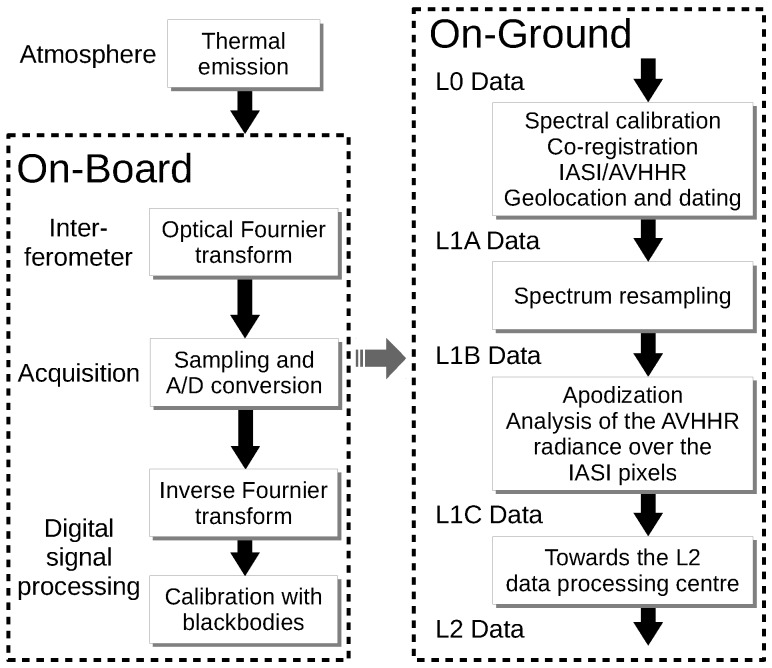
Main stages in the IASI data processing.

The data collected by the instrument are processed through an on-board and an on-ground processing chain until they are considered end-user products. The processing chain comprises different stages that yield products at various levels. The first data on ground are IASI L0 products: these have gone only through the on-board processing chain. The on-ground processing chain leads from IASI L0 to IASI L2 yielding intermediate products such as L1A, L1B and L1C. Figure 4 illustrates the main stages in the IASI data processing chain.

#### 2.3.1. On-Board Processing Chain

Data collected by the instrument are processed on board the satellite to produce calibrated atmospheric spectra from raw interferograms. The IASI data production rate is 45 Megabits/s, while the transmission rate allocated to IASI measurements is 1.5 Megabits/s. In order to reach the allocated data transmission rate, it is necessary to implement a significant part of the IASI data processing on board the satellite [51]. The main objective of the on-board processing chain is to convert raw interferograms into complex spectra meeting the allocated data rate to be transmitted to the ground station. Extensive details of the different processes performed by the instrument are described in [14,51].

#### 2.3.2. On-Ground Processing Chain

Once the data are received from the satellites, they are further processed until converted into end-user products. The on-ground processing chain comprises different stages, yielding products at different processing levels. L1A products are unapodized calibrated and geolocated spectra with corresponding Integrated Imaging Subsystem (IIS) images. L1B products are obtained from L1A after spectral resampling. L1C products are obtained from L1B after apodisation. In L1C level, the data are sampled every 0.25 cm${}^{-1}$ and the results of the analysis of Advanced Very High Resolution Radiometer (AVHRR) radiance over the IASI FOR are appended. The IASI L2 processing involves derivation of geophysical parameters from radiance measurements. This stage is performed in synergy with measurements from other instruments [50,52,53].

### 2.4. Data Dissemination

EUMETCast [54] is EUMETSAT’s primary dissemination mechanism for the near real-time delivery of satellite data and products. IASI products are thus mostly disseminated through EUMETCast to the NWP centres and scientific community. In turn, other institutions may request and distribute IASI data from EUMETSAT, for instance, the Physical Oceanography Distributed Active Archive Center (PODAAC) [55] and the Centre for Environmental Data Analysis (CEDA) [56] disseminate IASI L1C and L2 products.

In the case of EUMETCast, the number of registered users by June 2016 [57] (last available report) was over 4500 stations, with over 1,200,000 items delivered, and distributing more than 60 Terabytes (TB) per month. In the case of CEDA, 90.82 TB of IASI data were disseminated through 636,453 accesses during the last 12 months, and 466.74 TB through 1,188,507 accesses during the last 5 years [58].

Regarding IASI data, one of the most popular collections of distributed data is IASI Regional Data Service Level 1 [59], where 500 selected original IASI Channels and 300 Principal Component Scores (PCS) are combined in products with an average file size of 10 MB. These products discard many spectral channels and/or components due to the difficulties of transmitting files of larger size.

PCC is the common strategy used in the IASI community to reduce the large size of the data [60]. This technique is able to achieve a compression ratio of, approximately, 50:1 [16,61]. PCC is a lossy approach that reduces large correlated spectra, composed by thousands of channels, into some truncated pieces of information—the PCS—[62], which represent the most of the variance observed in the data. The most part of the atmospheric information is typically contained within the first few hundred of PCS, thus the most important information present in the spectrum can be preserved by retaining only the most significant PCS. The EUMETSAT Advanced Retransmission Service (EARS) provides a total of 290 PCS for the three bands of the IASI spectrum: 90 for Band 1, 120 for Band 2, and 80 for Band 3. This number of PCS allow to retain the atmospheric signal with negligible loss of information [16,63].

PCC exploits the high level of correlation between channels to achieve data compression [19]. The main advantage of PCC is the potential to remove part of the noise present in the original data [16]. However, atmospheric information is also lost. A reduction of the random instrument noise between 4 and 6 is achievable, while values of the reconstructed noise and the atmospheric information loss get close to the optimal ones proposed by the linear estimation theory [64].

A major concern in the use of PCC is that the PCS are determined from a training set. If the data used for training do not contain specific events, like volcanic eruptions, heavy biomass burning, wildfires, etc., these characteristics will not be present in the leading PCS and might be considered noise, reducing the usefulness of the data [62]. In order to minimize this drawback, a global training set, adequate to represent most of the atmospheric situations, should be employed and updated periodically to include rare events [61].

Another concern in the use of PCC is that some features associated with trace gases may not be properly retained in the reconstructed spectra, which is specially critical when the trace gas signal is weak. This may be caused when the number of PCS used in the reconstruction of the signal is not large enough or if the training set from which the reference eigenvectors were derived presents some deficiency [17,65].

PCC is a transform-based approach widely accepted for IASI dimensionality-reduction and for data compression, whose benefits and drawbacks are well known. Although IASI is not an imager but a sounder, coding techniques intended for images may also be employed to compress IASI data. In this paper we will analyze the performance of a wide range of coding techniques for lossless, near-lossless, and lossy compression of IASI data, including current standards and state-of-the-art coding techniques. We will review the performance of coding techniques that follow a different approach than PCC, such as prediction-based techniques, which allow lossless and near-lossess compression. We also evaluate transform-based coding techniques and the current video coding standard (HEVC), which include a rate-distortion optimisation stage to determine which contribution from each transformed channel should be included in the final compressed file, instead of applying the transform and selecting a subset of the transformed channels as PCC does.

We have observed in recent studies [26,27] that some of the proposed lossy compression schemes produce reconstructed radiances that are suitable for statistic retrieval algorithms, achieving competitive performance compared to retrievals performed over the original radiances.

One of the goals of our contribution is to report the performance of several compression schemes for IASI data, which allow different features in the reconstructed data as compared to PCC, for instance, compression of the whole spectra, specific accuracy in the recovered data, etc.

**Figure 5:**
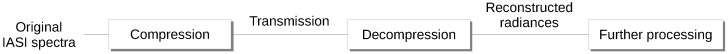
Sequential approach for IASI data compression.

Figure 5 illustrates the proposed coding approach. Once the original data (e.g., radiance data) have been compressed and transmitted, they must be decompressed to produce the reconstructed data (e.g., radiance data too). The dimensions and size of the reconstructed data are identical to that of the original data. If a near-lossless or a lossy compression paradigm is selected, the quality of the reconstructed data will be different than the quality of the original data.

IASI L1C products are the most common format for dissemination of IASI data [54,55,56,59] and will be the considered data in this research.

## 3. Data Compression

This section reports schematically the main characteristics of the compression techniques employed in this paper. Essentially, we introduce first the basic scheme of a data coding system, then we outline the skilled characteristics of the six examined coding techniques, next we provide the setting and parameters used for each tested coding technique, and finally we discuss the benefits of applying a spectral transform along the spectral dimension.

### 3.1. Data Coding System Pipeline

**Figure 6:**
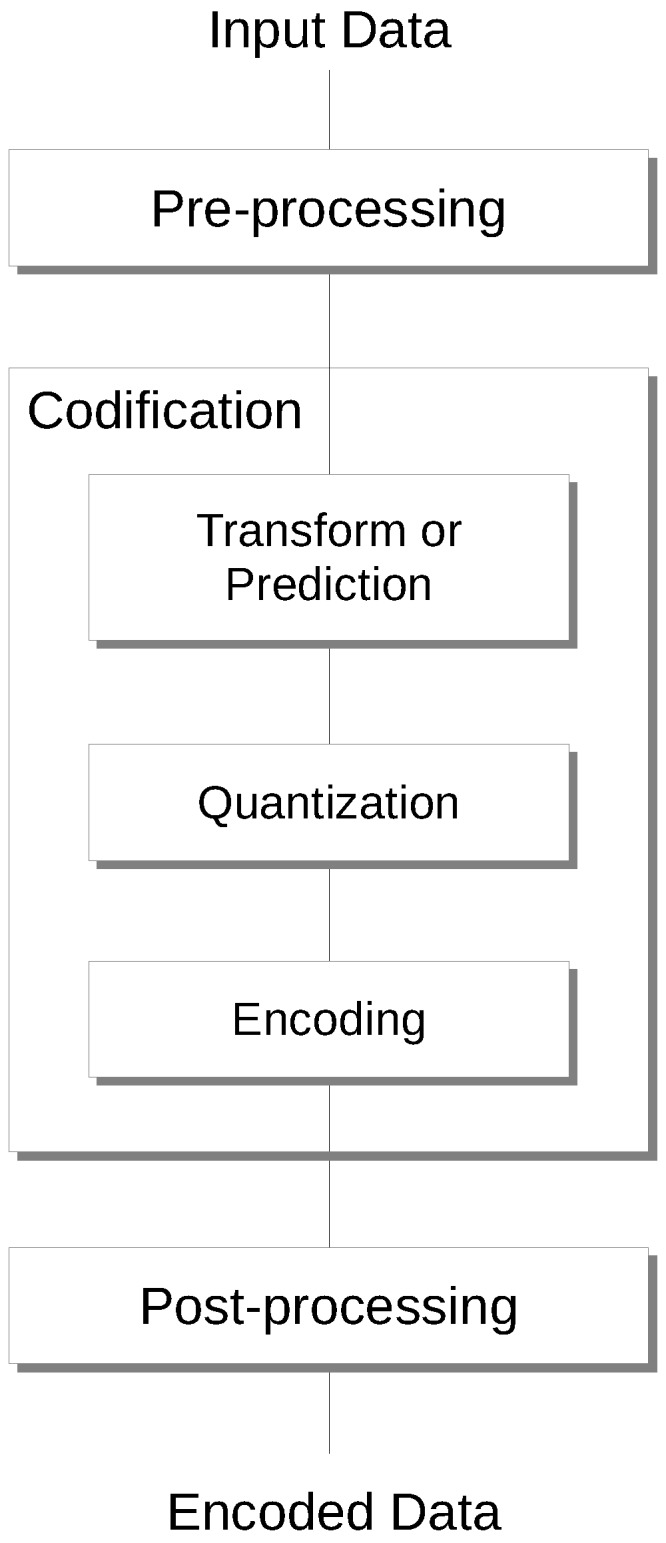
Data compression systems are usually composed of three main stages: pre-processing, coding, and post-processing. The coding stage may, in turn, comprise three steps: either transform or prediction, quantization, and encoding. Only the encoding process is displayed; decoding proceeds in reverse order.

A data coding system usually comprises three main stages: (1) pre-processing, (2) coding, and (3) post-processing, as illustrated in Figure 6.

The *pre-processing* stage is intended to prepare the data for the compression process. In some scenarios, like remote sensing, it may become a very important stage, having high influence in the later stages. Partitioning, denoising or segmentation are common processes performed during the pre-processing stage. The *coding* stage encodes the data resulting from the pre-processing stage. Different steps can be carried out in this stage. First, either a transform or a prediction step is applied to modify the representation space. The transform approach aims at providing a more decorrelated and compact representation of the signal. An example of this approach is the wavelet transform, providing a spatial-frequency domain representation. In its turn, the prediction approach aims at exploiting the correlation among neighbouring—causal—coefficients by guessing the next coefficient and incurring in a prediction error, which is expected to facilitate a better performing entropy encoding. The second step is a quantization step, applied in the case of near-lossless or lossy coding, as it entails a loss of information. The third step is an entropy encoding step. Common approaches include Huffman [36], Golomb [66] and Arithmetic encoding [67]. Depending on the compression technique employed, the *post-processing* stage can manipulate either the final codestream or the data recovered. In JPEG 2000, this stage organizes the final codestream to minimize the error between the original data and the reconstructed data at a desired target bit-rate. In HEVC, it defines some parameters for smoothing artifacts in the reconstructed data to improve its quality.

### 3.2. Characteristics of the Coding Techniques

Focusing on the coding stage, in this paper we screen two *transform-based* coding techniques, namely JPEG 2000 [30] and CCSDS-122.0 [32], three *prediction-based* coding techniques, namely JPEG-LS [29], M-CALIC [31] and CCSDS-123.0 [33], and the most recent video coding standard that includes both a transform and a prediction step, HEVC [34].

For each of the six considered compression techniques, Table 2 and Table 3 provide the following information: (1) *Year*: reports when that particular coding technique or standard was published; (2) *Compression paradigm*: indicates which of the three different coding paradigms are deployed, i.e., lossless, near-lossless, or lossy compression and whether that technique is prediction-based or transform-based; (3) *Reference*: cites the main reference; (4) *Pre-processing*: indicates what type of pre-processing stage is performed, if any; (5) *Post-processing*: indicates what type of post-processing stage is performed, if any; (6) *Spatial transform*: for the case of *transform-based* coding techniques, it provides information about what transform is employed to exploit the spatial redundancy; (7) *Prediction*: for the case of *prediction-based* coding techniques, it provides information about what type of prediction is employed to exploit redundancy and whether it is applied in the spatial direction (intra), in the spectral direction (inter), or in both; (8) *Quantization*: indicates what type of quantization is performed, if any; (9) *Bitplane encoding*: explains how a bitplane coding strategy [36] is applied, if any; and (10) *Entropy coder*: provides information about the type of entropy encoder used.

### 3.3. Setting and Parameter Configuration

All compression techniques allow different parameter and configuration options: on one hand, selecting appropriate settings has a significant influence on the compression performance; on another hand, these settings may determine the computational cost. Careful analysis has to be carried out to disclose appropriate settings.

To account for the reproducibility of the research, for the six evaluated coding techniques, and for each coding paradigm (as most coding techniques allow more than one coding paradigm), Table 4 provides the configurations of setting and mode, spatial transform and spectral transform used in our experiments. *Setting and mode* column refers to particular characteristics of each compression technique; *Spatial transform* column and *Spectral transform* column indicate what type of spatial or spectral transform is applied, if any.

Since JPEG-LS [29] and CCSDS-122.0 [32] coding techniques are devised to encode mono-channel data (2D data), for encoding data scenes with more than one channel (3D data) we used the following methodology: (1) split the data scenes into mono-channel data (in the case of IASI data, each spectral channel with a different wave-number shall be a mono-channel data), (2) each of those mono-channel data are individually encoded, and (3) the total bit-rate is the sum of the bit-rates for each mono-channel data. When JPEG-LS is paired with a spectral transform (see below), the scenes are first spectrally transformed and then the splitting procedure above is applied. In the case of CCSDS-122.0, the upcoming standard CCSDS-122.1 [68] is employed.

### 3.4. Spectral Transforms

To achieve competitive compression performance in hyperspectral data like IASI L1C, which are composed of more than 8000 channels, it is of paramount importance to exploit its high spectral redundancy. Spectral transforms are a commonly adopted approach. Among all the spectral transforms in the literature, we focused on four of them: (1) Wavelet Transform (WT), due to its extended use and low complexity; (2) the Karhunen-Loève Transform (KLT), because it is the optimal decorrelating transform for gaussian sources despite its high computational cost; (3) the Pairwise Orthogonal Transform (POT), as it is a low complexity approximation to KLT and is at the basis of upcoming standard CCSDS-122.1; and (4) the Regression Wavelet Analysis Transform (RWA) because of its highly competitive performance and bearable computational cost.

Depending on the coding paradigm used, a reversible (RKLT, RPOT, IWT 5/3, RWA) or an irreversible (KLT, POT, DWT 9/7) version of the spectral transforms must be employed. For RWA, two different estimation models [38] could be adopted: *Maximum model* and *Restricted model*. Here, we use a variant of the Maximum model, the *Exogenous variant*, which considerably reduces the computational cost and does not entail transmission of any side-information.

All of these spectral transforms are used in combination with the six coding techniques evaluated here. Although M-CALIC, CCSDS-123.0 and HEVC already exploit themselves the spectral redundancy by employing prediction techniques, we also pair them with the different spectral transforms and evaluate their coding performance.

**Figure 7:**
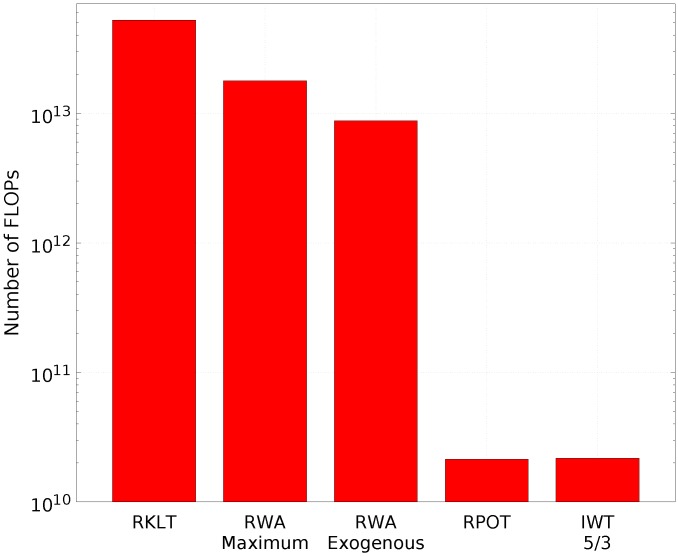
Cost comparison in FLOPs for the different spectral transforms used in the experiments applied to an IASI L1C orbit with 8461 spectral channels and a spatial resolution of 765×30×4 (number of scan lines × number of FORs per line × number of IFOVs per FOR).

A particular aspect to consider when applying a spectral transform is the computational complexity, because it may be critical in many scenarios. The computational cost in floating-point operations (FLOPs) of applying lossless forward and backward transforms on a typical IASI L1C orbit for RKLT, IWT, RPOT, and RWA is shown in Figure 7. In this particular case, the RKLT cost is over 2400 times higher than that of RPOT or IWT, and approximately 6 times higher than that of Maximum RWA (Exogenous variant).

Table 5 reports a detailed analysis for the reversible version of each spectral transform, in terms of computational cost (in FLOPs). The computational cost of RKLT and Multilevel Clustering RKLT (see below) mainly depends on the squared number of spectral channels, which substantially increases the computational cost as the number of channels increases. The IWT 5/3 and RPOT transforms have an approximately linear cost in relation to the spatial locations and the spectral channels. The cost of RWA is dominated by the estimation of the regression coefficients and the generation of the predictions [38]. In the case of the Exogenous variant, the estimation stage is performed offline and does not imply additional transform cost during the encoding process.

As seen, a case of very high computational cost and memory requirements transform is KLT/RKLT, which renders it unusable in situations where the number of spectral channels is large. To alleviate its high computational cost, there exist a number of strategies. Here, we use a divide-and-conquer strategy, the Multilevel Clustering KLT/RKLT [69], as described in Section 3.5.

Some spectral transforms may produce data with more than 16 bits per pixel per channel (bpppc), whereas software implementations used for JPEG-LS, M-CALIC, CCSDS-122.0, CCSDS-123.0, and HEVC deal with input data of at most 16 bpppc. In these cases, each transformed channel is split into two different channels: a channel formed by the 16 most significant bits (MSB), and a channel formed by the 16 least significant bits (LSB). The MSB channels and the LSB channels are grouped in two different volumes and encoded separately. The total bit-rate of the compressed data is the sum of the bit-rates of the two compressed volumes.

### 3.5. Divide-and-Conquer Strategy for KLT/RKLT

**Figure 8:**
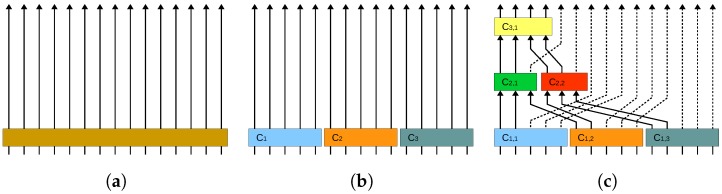
Structure of plain KLT/RKLT, Classical Clustering KLT/RKLT, and Multilevel Clustering KLT/RKLT. This example decorrelates 15 spectral channels. Each arrow denotes a channel and each coloured rectangle represents the computation of a KLT/RKLT transform. In the case of Classical Clustering KLT/RKLT, three clusters are employed. In the case of Multilevel Clustering KLT/RKLT, 3 levels of Multilevel Clustering are applied. (**a**) Plain KLT/RKLT; (**b**) Classical Clustering KLT/RKLT; (**c**) Multilevel Clustering KLT/RKLT.

The Classical Clustering divide-and-conquer strategy divides a large transform in several clusters and applies a smaller transform to each cluster. This approach significantly reduces the overall computational cost, but only provides local decorrelation within each cluster. Global decorrelation can be achieved by applying a Multilevel Clustering strategy, where the most important parts of each local transform are further decorrelated in the next levels. In Figure 8, the structure of a plain KLT/RKLT transform, of a Classical Clustering KLT/RKLT and of a Multilevel Clustering KLT/RKLT are displayed.

The computational complexity of KLT/RKLT stems from the number of spectral channels to be transformed. The complexity of Multilevel Clustering KLT/RKLT depends thus on the employed cluster size. An appropriate configuration for applying Multilevel Clustering KLT/RKLT on IASI L1C orbits is found by assessing three different criteria: *computational cost*, related to the *execution time*, and *transform coding performance*. To perform this assessment, we consider an IASI L1C orbit with $2^{13}$ (8192) spectral channels (discarding the last 269 channels of the 8461 channels spectrum).

#### 3.5.1. Computational Cost

**Figure 9:**
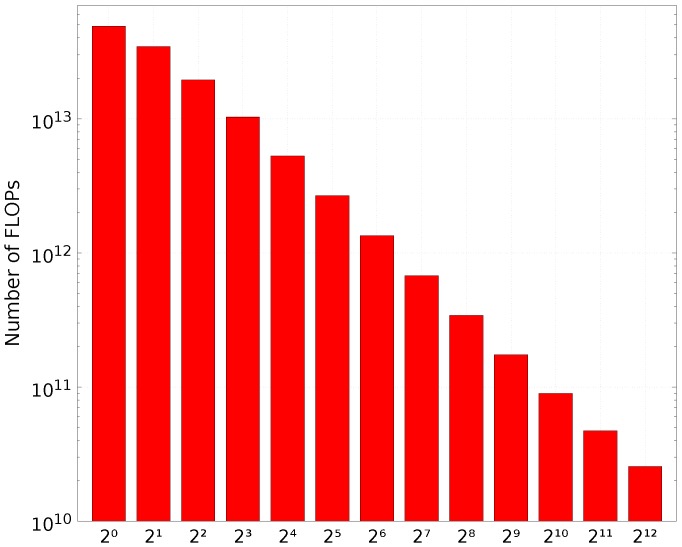
Cost comparison in FLOPs for different cluster sizes of Multilevel Clustering RKLT applied to an orbit with $2^{13}$ spectral channels and a spatial resolution of 765×30×4 (number of scan lines × number of FORs per line × number of IFOVs per FOR).

The computational cost of different cluster sizes for Multilevel Clustering RKLT is illustrated in Figure 9. The computational cost rapidly increases as the number of clusters defined in the first level decreases, which is equivalent to increase the cluster size. Notice that using 1 cluster is identical to not using any clustering strategy.

#### 3.5.2. Execution Time

**Figure 10:**
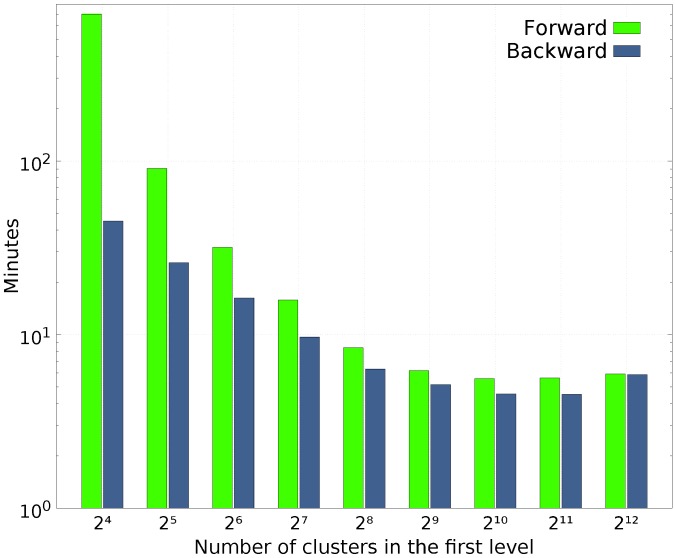
Runtime comparison in minutes for different cluster sizes of Multilevel Clustering RKLT applied to an IASI L1C orbit with $2^{13}$ spectral channels and a spatial resolution of 765×30×4 (number of scan lines × number of FORs per line × number of IFOVs per FOR). The dissemination granularity of the data is 3 min for Level 1c [70].

The forward and backward execution times of different cluster sizes when Multilevel Clustering RKLT is applied to the proposed $2^{13}$ IASI L1C orbit are compared in Figure 10. The longest runtimes are required when less than $2^{5}$ clusters ($2^{8}$ channels per cluster) are defined in the first level. Execution times are not provided for 1, 2, 4, and 8 clusters defined in the first level due to the high computational complexity. All experiments have been performed on an Intel Xeon CPU E3-1230 V2 @ 3.30 GHz processor.

#### 3.5.3. Transform Coding Performance

The performance of Multilevel Clustering RKLT in terms of both computational cost and transform coding performance is illustrated in Table 6. The best trade-off between computational cost and entropy of the transformed orbit is obtained when $2^{7}$ (128) or $2^{8}$ (256) clusters are defined in the first level.

Based on the previous analysis of Multilevel Clustering RKLT, 200 clusters in the first level and multilevel mode has been used in all the experiments of this manuscript.

For the irreversible case, a Multilevel Clustering KLT with the same configuration has been selected too, because the coding performance for KLT and Multilevel Clustering KLT is almost equivalent, because Multilevel Clustering KLT requires much less side-information than KLT, and because although KLT has a lower computational complexity than RKLT, its application on a 8461 IASI L1C orbit may take over 30 h.

## 4. Experimental Results

This section presents a set of experiments aimed at the analysis and evaluation for lossless, near-lossless, and lossy compression of IASI L1C products. First, a description of the IASI L1C products and the software employed in the experiments is provided. Then, we will focus on the compression results produced by the different coding techniques.

### 4.1. Data Collection and Software

To obtain sound conclusions, the experiments are conducted with a set of 96 IASI L1C orbits granted by EUMETSAT (http://catalogue.ceda.ac.uk/), representing more than 148 Gigabytes. 48 orbits belong to IASI-A and 48 orbits belong to IASI-B. These orbits are acquired with, respectively, MetOp-A and MetOp-B satellites. To get a representative data set, orbits acquired throughout a full year are selected for each sensor: from July 2013 to June 2014, 4 orbits per month, 1 per week. For the sake of conciseness, details and results will be grouped by instrument, computing the average of the 48 orbits. Results for each individual orbit are very similar. All data are 16 bpppc and are stored as signed integers. For each product, Table 7 provides the sizes and the average zero-order entropy, which is the smallest number of bits, on average, required to represent a sample without considering any dependency among pixels within or between channels.

All software used to produce the experimental results is public. The implementations employed are the following: JPEG-LS software [71], Kakadu software [72] for JPEG 2000, M-CALIC software [73], TER software [74] for CCSDS-122.0, EMPORDA software [75] for CCSDS-123.0, and HEVC software [76]; Spectral Transform software [77] for Multilevel Clustering KLT/RKLT and WT, Pairwise Orthogonal Transform software [78] for POT/RPOT, and Regression Wavelet Analysis software [79] for RWA.

### 4.2. Lossless Compression Results

Lossless compression of IASI L1C products is evaluated for the suggested approach: spectral transform followed by coding technique. Four spectral transforms have been tested: Multilevel Clustering RKLT, IWT, RPOT and RWA. All six coding techniques are assessed: JPEG-LS, JPEG 2000, M-CALIC, CCSDS-122.0, CCSDS-123.0 and HEVC. Table 8 reports the average lossless coding performance (compression ratio). Results suggest that:

- *Coding performance for IASI-A and IASI-B products is nearly the same.* Lossless compression of IASI-B products is, on average, only 0.75% better than for IASI-A. This negligible difference happens for all IASI-A and IASI-B products and for all compression schemes.
- *IASI L1C data present high spectral redundancy.* M-CALIC, CCSDS-123.0 and HEVC, which originally exploit the spectral redundancy, achieve better outcomes than JPEG-LS, JPEG2000 or CCSDS-122.0, which do not exploit this redundancy. For the latter techniques, taking advantage of this redundancy through a spectral transform yields significantly better compression performance, bridging the gap with the former techniques.
- *Compression techniques that already exploit the spectral redundancy by themselves also benefit from applying a spectral transform.* When paired with a spectral transform, M-CALIC, CCSDS-123.0, and HEVC usually achieve better coding performance too (except for IWT + M-CALIC and RPOT + CCSDS-123.0). This effect is specially significant in the case of HEVC, where up to 11.11% can be improved, but also for M-CALIC, where gains are close to 9%. Gains for CCSDS-123.0, which was the coding technique providing the best performance, are less meaningful.
- *Multilevel Clustering RKLT or RWA yield the best coding performance.* Multilevel Clustering RKLT brings the largest improvements, closely followed by RWA. As compared to original CCSDS-123.0, which is the coding technique providing the best performance when no spectral transform is applied, the improvements for Multilevel Clustering RKLT and for RWA when combined with M-CALIC are, respectively, of 4.7% and 2.4%.
- *Compression ratios over 2.5:1 (bit-rates close to 6.3 bpppc) can be achieved for lossless compression of IASI L1C products.* The best results are obtained by *Multilevel Clustering RKLT + M-CALIC*, which achieves, on average, a compression ratio of 2.54:1 for IASI-A products and 2.56 for IASI-B products.

### 4.3. Near-Lossless Compression Results

Two different coding techniques, M-CALIC and JPEG-LS, are used for near-lossless compression of IASI L1C products. Eight different Peak Absolute Errors (PAE) (δ∈{1,3,7,15,31,63,127,255}) are considered. Results are reported in Table 9. Three main observations can be drawn:

- *As expected, compression ratio increases as PAE increases.*
- *Competitive compression performance is achieved even by allowing small errors.* Large savings over 17% and 30% with respect to lossless compression are already achieved for such small PAE as 1 and 3.
- *M-CALIC yields higher compression ratio than JPEG-LS.* M-CALIC uses an arithmetic coder, while JPEG-LS uses Golomb codes, for which bit-rates below 1 bpppc are not achievable.

**Figure 11:**
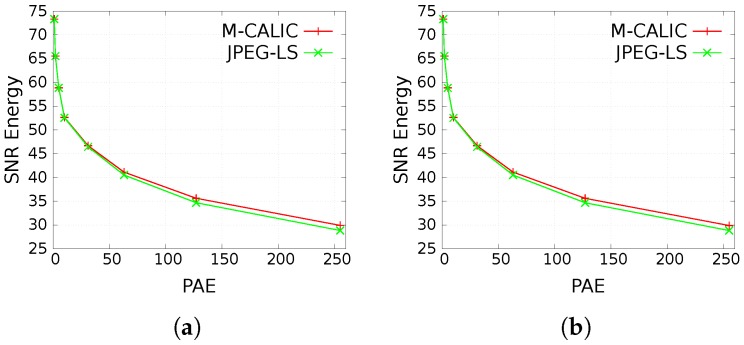
Rate-distortion performance of near-lossless compression of IASI L1C products. Results report SNR Energy (in dB, higher is better) vs. PAE. (**a**) IASI-A; (**b**) IASI-B.

Figure 11 illustrates the rate-distortion performance of near-lossless compression in terms of Signal Noise Ratio (SNR) Energy vs. PAE. Using as small PAE as 1 and 3, SNR Energy over 65 dB can already be achieved.

### 4.4. Lossy Compression Results

Lossy compression of IASI L1C products is evaluated using JPEG 2000 and CCSDS-122.0 standards along with three spectral transforms: Multilevel Clustering KLT, DWT and POT. All schemes are evaluated using nine target bit-rates between 0.01 and 2 bpppc.

**Figure 12:**
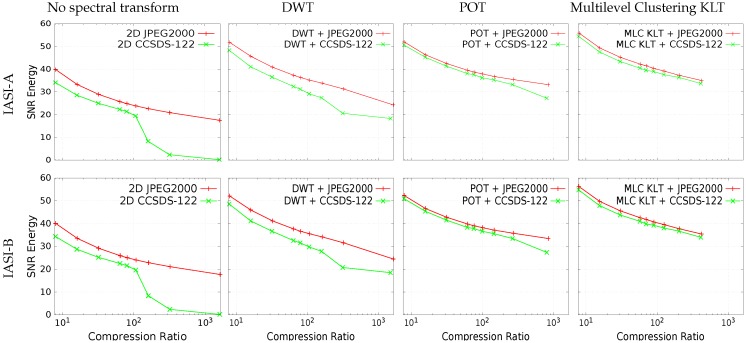
Rate-distortion performance of lossy compression of IASI L1C products. Results report SNR Energy (in dB, higher is better) vs. compression ratio. Results for different spectral transforms are plotted in the columns. In each plot, curves for JPEG 2000 and CCSDS-122.0 performance are displayed. Ranges are the same in all the plots to ease the comparison. Top row: IASI-A products; Bottom row: IASI-B products. POT and Multilevel Clustering KLT are not able to reach such high compression ratios (over 1000:1) as DWT because side-information needs to be transmitted besides the compressed data.

Figure 12 illustrates the lossy compression performance of IASI L1C products for JPEG 2000 and CCSDS-122.0. Several conclusions can be drawn:

- *Exploiting the spectral redundancy is essential to achieve competitive performance.* Applying a spectral transform always outperforms the scheme that does not exploit the spectral redundancy. Performance difference is more apparent as the compression ratio decreases, growing from 5 to over 15 dB.
- *Multilevel Clustering KLT yields the best coding performance.* As happened for lossless compression, also in the case of lossy compression, Multilevel Clustering KLT furnishes the highest results, followed by POT and DWT. At high compression ratios (higher than 20:1), POT yields almost equivalent performance, mostly because of the larger size of the side-information needed by Multilevel Clustering KLT.
- *JPEG 2000 outperforms CCSDS-122.0.* JPEG 2000 is a more complex coding technique that is able to produce more competitive results.
- *Plain 2D CCSDS-122.0 yields low performance at high compression ratios.* This standard starts achieving good results for compression ratios lower than 100:1.

### 4.5. Comparison between Near-Lossless and Lossy Compression

**Figure 13:**
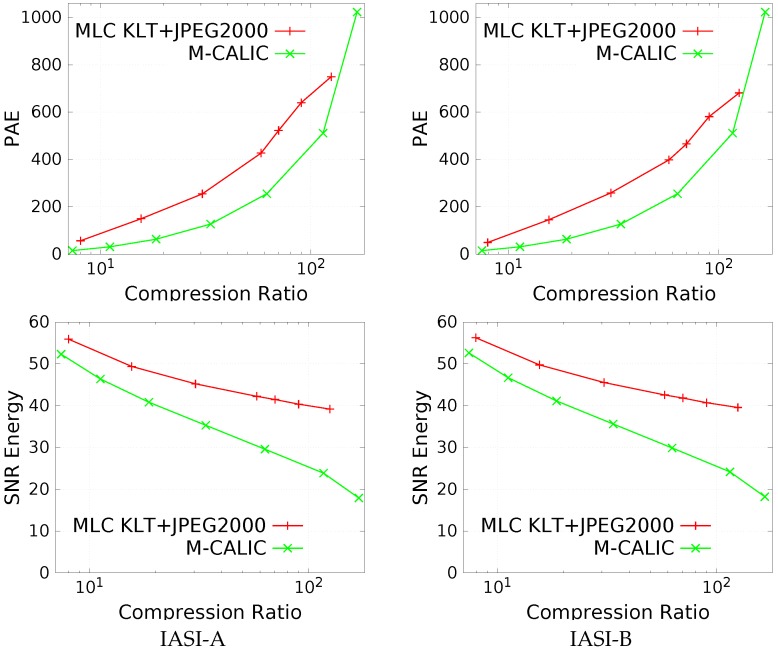
Performance comparison between near-lossless (M-CALIC) and lossy compression (Multilevel Clustering KLT + JPEG 2000). Top row: PAE (lower is better); Bottom row: SNR Energy (in dB, higher is better).

A comparison of the two analyzed compression paradigms that introduce distortion in the reconstructed data, i.e., near-lossless and lossy compression, is performed in Figure 13. The best coding scheme for near-lossless (M-CALIC) and for lossy (Multilevel Clustering KLT + JPEG 2000) compression are compared from the point of view of PAE and SNR Energy. Bit-rates between 0.1 and 2 bpppc are compared (very large PAE—higher than 1023—are requested to achieve bit-rates lower than 0.1 for near-lossless compression). Some conclusions can be drawn:

- *Near-lossless outperforms lossy compression in terms of PAE.* Near-lossless compression introduces lower maximum errors in the data than lossy compression.
- *Lossy compression outperforms near-lossless compression in terms of SNR Energy.* Lossy compression yields larger results, especially at large compression ratios.

### 4.6. Compression and Decompression Runtimes

IASI Level 1 products are distributed to users in different dissemination modes and formats. While the timeliness for Near-Real Time dissemination through EUMETCast is 2 h 15 min, the timeliness for products on the EUMETSAT Data Centre retrieval is approximately 8–9 h [70]. Table 10 summarizes the compression runtimes for the coding schemes that provide the best performance for lossless, near-lossless and lossy compression. The decompression runtimes, which are applicable at the receiver side, are also provided. All experiments have been performed on an Intel Xeon CPU E3-1230 V2 @ 3.30 GHz processor.

Compression schemes that involve lossy coding achieve competitive runtimes and might be considered in a near-real time scenario. Both near-lossless and lossy compression require less than 15 min in the compression stage. At the receiver side, 11 and 6 min for near-lossless and lossy coding, respectively, would be required to decompress the codestream. Longer runtimes are required for lossless compression mainly due to the computation of Multilevel Clustering RKLT. Lossless compression would be appropriate only in scenarios where the delivery time is not critical.

### 4.7. Analysis of the Reconstructed Radiances

To evaluate the usefulness of the reconstructed radiances, M-CALIC and Multilevel Clustering KLT + JPEG 2000, which are the compression schemes that produce the best performance for, respectively, near-lossless and lossy compression, are compared with Principal Component Compression (PCC).

The experiments are conducted using the product IASI_xxx_1C_M02_20140305023859Z_20140305042058Z_N_O_20140305042027Z (details about file naming convention can be found at [50]). To simplify the comparison, the first 1800 channels of this IASI L1C orbit are used. All of them belong to Band-1. Two compression ratios are compared by retaining a different number of eigenvectors in PCC, either 150 or 200, which is common in practical scenarios. For M-CALIC and Multilevel Clustering KLT + JPEG 2000, the PAE and target bit-rate that produce, respectively, the same compression ratio as compared to PCC are employed. Table 11 summarizes the settings for each experiment.

**Figure 14:**
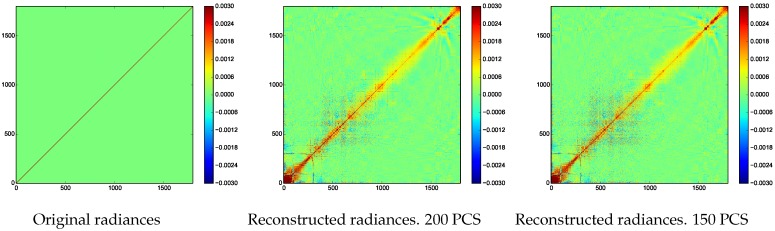
Noise covariance matrix of the original radiances and noise covariance matrix of the reconstructed radiances after Principal Component Compression when 200 and 150 PCS are employed.

The noise covariance matrix of the original radiances and of the reconstructed radiances after Principal Component Compression for experiment 1 and experiment 2 are illustrated in Figure 14. It is known that the noise covariance matrix of the original radiances is diagonal, while the noise covariance matrix of the reconstructed radiances is quite similar when 200 or 150 PCS are employed.

**Figure 15:**
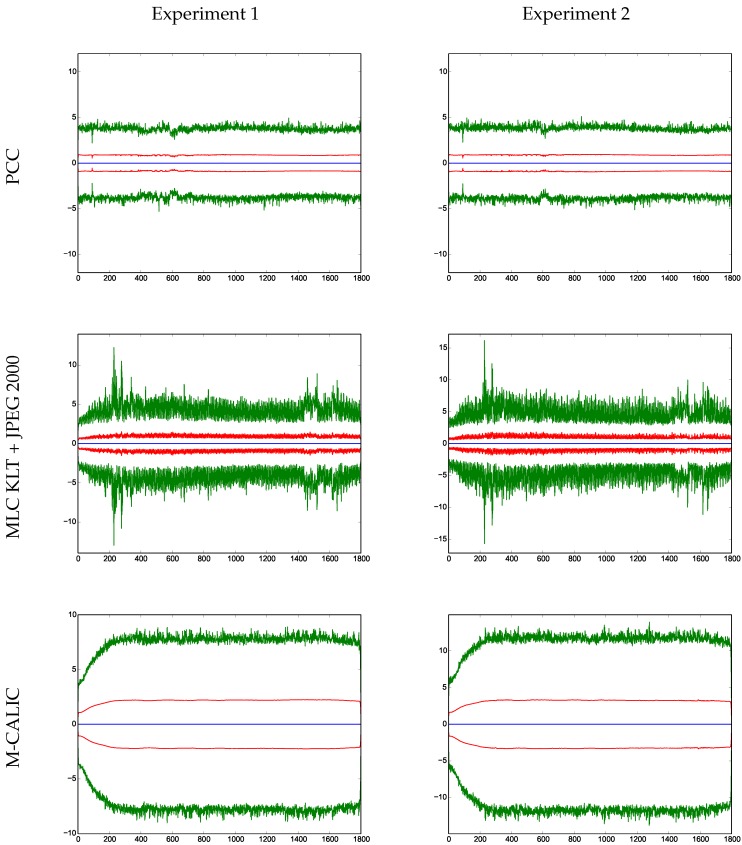
Normalized radiance residuals statistics. The average of the normalized radiance residuals is shown in blue, standard deviation in red, and maximum and minimum values in green.

In Figure 15 the normalized radiance residual statistics as a function of component number for experiment 1 and experiment 2 are shown. The normalized reconstructed radiances are subtracted from the normalized original radiances. Normalization takes into account the noise covariance matrix inherent to IASI. The average of the normalized radiance residuals, the standard deviation and the maximum and minimum values per channel are reported.

The average of the normalized radiance residual and the standard deviation are very similar for all compression schemes. As for the maximum and minimum differences, PCC and Multilevel Clustering KLT + JPEG 2000 produce smaller values as compared to M-CALIC. The magnitude of the maximum and minimum values is slightly lower for PCC than for Multilevel Clustering KLT + JPEG 2000.

**Figure 16:**
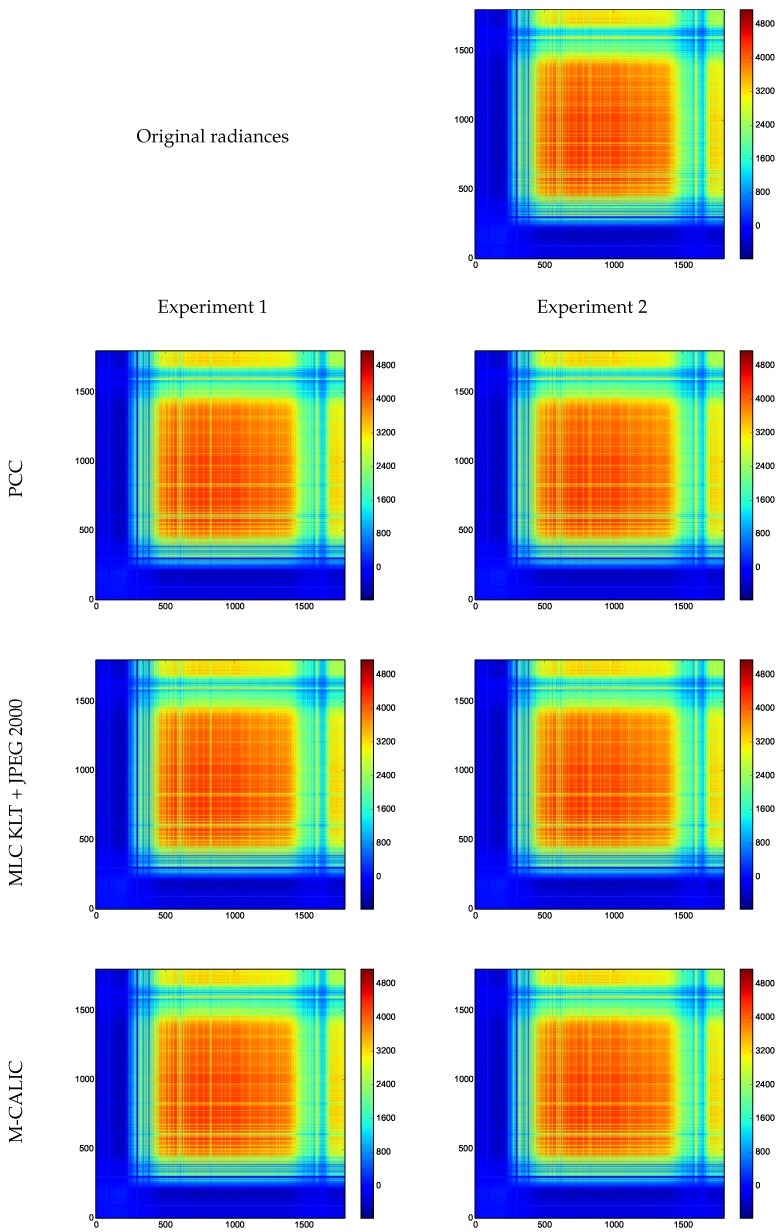
Covariance matrix of the original radiances and covariance matrix of the reconstructed radiances.

The covariance matrix of the original and reconstructed radiances is illustrated in Figure 16 for the three different coding techniques at the proposed compression ratios. The nature of the original data and of the reconstructed data has a similar nature.

**Figure 17:**
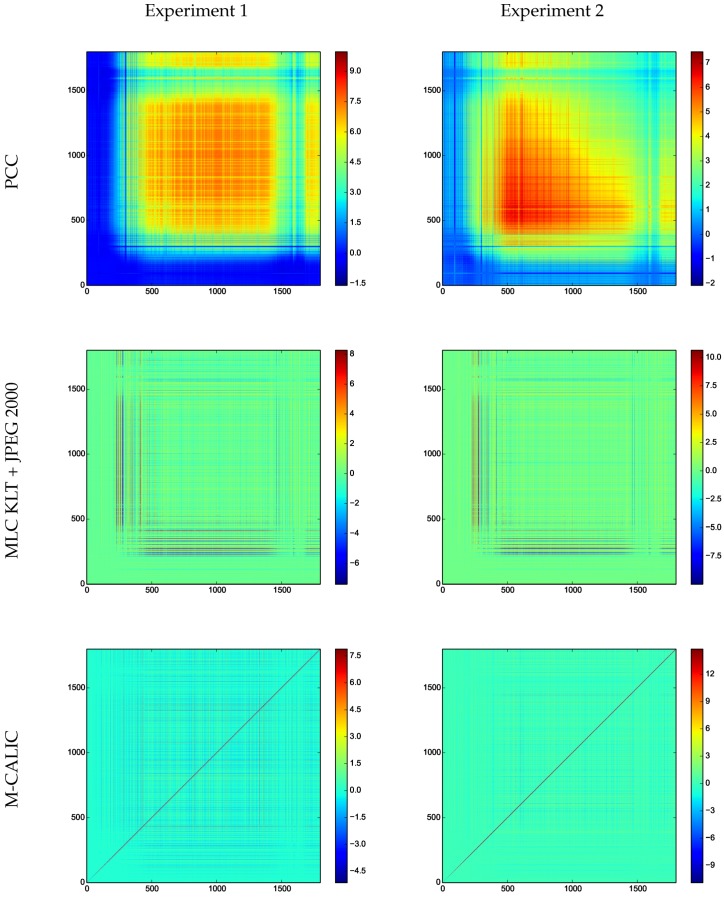
Differences between the covariance matrix of the original radiances and the covariance matrix of the reconstructed radiances.

To analyze the impact of the compression, Figure 17 reports the differences between the covariance matrix of the original radiances and the covariance matrix of the reconstructed radiances.

The differences are very similar for the two compression ratios analyzed in all coding schemes. The covariance matrices of the reconstructed spectra from Multilevel Clustering KLT + JPEG 2000 and M-CALIC are very similar to the covariance matrix of the original data. For M-CALIC, the difference is focused in the main diagonal, while for Multilevel Clustering KLT + JPEG 2000 the differences are clear in a small set of channels. For PCC, the differences are more apparent.

### 4.8. Discussion

The performance of IASI L1C data compression has been investigated for lossless, near-lossless, and lossy compression. Lossless compression is sometimes a demanded requirement in remote sensing applications because introducing some amount of distortion in the reconstructed data may compromise the quality of derived products. For IASI orbits, lossless compression can contribute to alleviate the large size of the data. As reported in Table 8, lossless compression can reduce the data size to less than half the original size, achieving compression ratios of 2.5:1.

Experimental results reveal that compression techniques that originally exploit the spectral redundancy such as M-CALIC, CCSDS-123.0 and HEVC produce better performance than JPEG-LS, JPEG2000 and CCSDS-122.0, which do not exploit the spectral dimension. It is acknowledged that exploiting the spectral redundancy present in hyperspectral data is of paramount importance to achieve competitive compression performance [80]. This is especially critical in the case of IASI L1C products due to the large number of highly correlated spectral channels. CCSDS-123.0, which is the coding technique providing the best compression performance, is superior to JPEG-LS, JPEG 2000, and CCSDS-122.0 in 26.5%, 28.5%, and 30.5%, respectively. When JPEG-LS and JPEG 2000 are paired with a spectral transform to exploit the spectral redundancy, they surpass CCSDS-123.0. For CCSDS-122.0 prepended with a spectral transform, the difference with respect to CCSDS-123.0 decreases to less than 4%.

However, the large spectral dimension of IASI data imposes a careful selection to exploit the spectral redundancy. Some spectral transforms, such as RKLT, may be unusable when the number of spectral channels is large due to its expensive computational complexity. In this case, a divide-and-conquer strategy like Multilevel Clustering RKLT may be a very effective approach [81], both in terms of coding performance and of computational cost. If lower computational requirements are demanded, RPOT and IWT are two alternatives, although they yield lower compression performance. RWA spectral transform provides also a competitive coding performance, improving on average, 7.1% and 6.9% with respect to RPOT and IWT.

We observed that compression techniques that already exploit the spectral redundancy by themselves also improve the coding performance when a spectral transform is applied. This is due to the large spectral dimension and the high redundancy present in IASI data. For HEVC and M-CALIC up to 11.11% and 8.59%, respectively, can be improved. In the case of CCSDS-123.0, gains are less significant.

Lossless compression can be an appropriate approach to compress IASI L1C products because all information is preserved, however the achieved compression ratios are limited. When larger compression ratios are requested, near-lossless or lossy compression is needed. Although using these approaches prevents a perfect reconstruction, it has been observed that some applications do not reduce their performance when certain level of distortion is introduced [26,27]. Near-lossless and lossy compression might be two reasonable compression approaches for IASI L1C products. In fact, data disseminated today through IASI Regional Data Service is not either the complete original scene, since only 800 (or less) out of the original 8461 channels are distributed for near-real time dissemination.

If a specific accuracy must be preserved in the reconstructed data, near-lossless compression is a proper strategy since the quality of the recovered data can be controlled by bounding the peak absolute error per pixel. In our experiments, we observed that using as small PAEs as 1 and 3, compression ratios of, respectively, 3:1 and 4:1 can already be achieved, while the data quality, measured in SNR Energy, still exceeds 65 dB. Table 9 illustrates how the compression ratio increases as the allowed PAE increases. Among the two compression techniques evaluated for near-lossless compression, M-CALIC produces larger compression ratios than JPEG-LS, achieving similar SNR Energy. The performance improvement of M-CALIC increases as PAE value increases, being, approximately, 25% more competitive for small PAEs and 70% for large PAEs. M-CALIC produces improved performance mainly due to the ability of M-CALIC to exploit the spectral redundancy present in the data, which is essential to achieve competitive coding performance. JPEG-LS is a 2D data compression standard and is not able to exploit the spectral dimension. In addition, M-CALIC implements an arithmetic coder, while JPEG-LS uses Golomb codes, not being able to produce bit-rates below 1 bpppc. In this case, pairing JPEG-LS with a spectral transform would not be an appropriate approach, because the spectral transform forestalls the precise control over the peak absolute error.

For lossy compression, as happened for lossless compression, exploiting the spectral redundancy yields improved outcomes. Experimental results reveal that applying a spectral transform always produce better performance, with differences increasing for smaller compression ratios, growing from 5 to over 15 dB. Multilevel Clustering KLT also produces always the best coding performance among the spectral transforms. Applying POT achieves similar performance at compression ratios higher than 20:1, mainly due to the larger size of the side-information produced by Multilevel Clustering KLT. In turn, employing POT produces more competitive performance than DWT at large compression ratios, but the performance gets closer at small compression ratios.

With regard to comparison between coding standards JPEG 2000 and CCSDS-122.0, the former always yields improved coding performance, for both lossless and lossy. When paired with Multilevel Clustering KLT or POT spectral transforms, these two coding techniques show very similar behaviour for lossy compression, while there is a difference of about 5.5% for lossless.

Applying a compression process through either M-CALIC or Multilevel Clustering KLT + JPEG 2000, the compression schemes that produce the best coding performance for, respectively, near-lossless and lossy compression, takes less than 15 min, which may be acceptable in a near-real time scenario. The decompression stage, required to retrieve the reconstructed spectra at the receiver side, takes, approximately, 11 and 6 min for, respectively, near-lossless and lossy compression. Multilevel Clustering RKLT, the lossless scheme that produces the largest compression ratios, requires longer runtimes mainly due to the application of the Multilevel Clustering RKLT.

The analysis of the recovered data indicated that the covariance matrix of the reconstructed radiances for both Multilevel Clustering KLT + JPEG 2000 and M-CALIC is very similar to the covariance matrix of the original radiances. Although promising, more experiments are needed to determine whether these two coding schemes could become an alternative to Principal Component Compression for IASI data near-real time dissemination.

Finally, we note that the compression performance for IASI-A and IASI-B products is almost equivalent for lossless, near-lossless, and lossy compression.

## 5. Concluding Remarks

Infrared Atmospheric Sounding Interferometer (IASI) data acquired from MetOp-A and MetOp-B satellites are mostly disseminated as IASI L1C products. These products have provided over 10 years of continuous observations, and with the foreseen launch of MetOp-C satellite in October 2018, this long time series of climate variables will be further extended. So far, distribution of IASI L1C data has been mostly conducted through IASI Regional Data Service Level 1, where 800 (or less) channels out of the original 8461 spectral channels are disseminated in near-real time, however, a number of applications have recently identified the need to operate with the complete range of spectral channels. Transmission and storage of complete scenes with such large size pose a challenge, which might be alleviated thanks to data compression.

In this paper we put forward a comprehensive study of IASI L1C data compression. Lossless, near-lossless and lossy compression paradigms have been investigated on a representative set of 96 orbits selected over a full year, 48 orbits from each MetOp-A and MetOp-B satellite, 4 orbits per month. Two wavelet-based coding standards, JPEG 2000 and CCSDS-122.0, three prediction-based techniques, JPEG-LS, M-CALIC and CCSDS-123.0, and the most recent video coding standard, HEVC, have been evaluated. To account for the large spectral redundancy in IASI products, four spectral transforms, RKLT/KLT, IWT/DWT, RPOT/POT and RWA, have been combined with the six coding techniques and their performance assessed.

Experimental results suggest that Multilevel Clustering RKLT/KLT is an efficient approach in terms of both coding performance and computational complexity, providing the best outcome for lossless and lossy compression when paired with, respectively, M-CALIC and JPEG 2000. For near-lossless compression, M-CALIC is the best performing technique.

The covariance matrix of the reconstructed radiances for Multilevel Clustering KLT + JPEG 2000 and M-CALIC, the compression schemes that provides the best coding performance for lossy and near-lossless compression, respectively, are very similar to the covariance matrix of the original radiances, which suggests that the quality of the recovered data is still adequate for further processings. Although promising, more experiments are needed to determine whether the proposed compression schemes could become an alternative to Principal Component Compression for IASI data near-real time dissemination.

The reported analysis can contribute to deploy new methodologies to manage data from current and upcoming high spectral resolution infrared instruments and improve the quality of disseminated products as demanded in several application areas. It is important to note that the selected compression scheme must preserve the atmospheric information content and reduce the level of noise contained in the data, while achieving competitive compression ratios.

## Figures and Tables

**Table 1:** Main characteristics of IASI instrument [50].

Characteristics of IASI instrument	
Orbit	Polar sun-synchronous
Time for one orbit	101 min
Global Earth coverage	2 times per day
Repeat cycle	29 days (412 orbits)
Altitude	∼819 km
Scan type	Step and stare
Interferograms	30 per scan line
	151 ms per interferogram
	taken in equally spaced time intervals every 8/37 s
FOR	30 per line
	50 km (3.33°) at nadir position
	4 simultaneous IFOVs of 12 km
Full swath width	∼2200 km (±48.3°)
Data production	120 spectra every 8 s
	∼1,300,000 observations per day
Data acquisition rate	45 Mbps
Data transmission rate	1.5 Mbps
Spectral range	Band-1: 645–1240 cm${}^{-1}$
	Band-2: 1200–2040 cm${}^{-1}$
	Band-3 :1960–2760 cm${}^{-1}$
Spectral sampling	0.25 cm${}^{-1}$ (0.5 cm${}^{-1}$ apodized)

**Table 2:** Technical characteristics of the considered compression techniques (year, compression paradigm, reference, pre-processing, and post-processing).

	JPEG-LS	JPEG 2000	M-CALIC	CCSDS-122.0	CCSDS-123.0	HEVC
Year	1999	2000	2004	2005	2012	2013
Compression Paradigm	Lossless and near-lossless	Lossless and lossy	Lossless and near-lossless	Lossless and lossy	Lossless	Lossless and lossy
	Prediction-based	Transform-based	Prediction- based	Transform-based	Prediction-based	Prediction- and Transform-based
Reference	[29]	[30]	[31]	[32]	[33]	[34]
PRE-PROCESSING						
	✗	Possibility of multi-channel transform, tile partitioning, and level-shift for unsigned data	✗	✗	✗	Possibility of tiles. channels are partitioned into Coding Tree Units (CTUs).
POST-PROCESSING						
	✗	Bit-stream organization (bit-allocation, data ordering, error resilience, and file format)	✗	✗	✗	Deblock Filtering (DBF) and Sample-Adaptive Offset (SAO). Both stages are optional.

**Table 3:** Technical characteristics of the considered compression techniques (coding).

	JPEG-LS	JPEG 2000	M-CALIC	CCSDS-122.0	CCSDS-123.0	HEVC
CODING						
Spatial transform	✗	Wavelet transform (up to 32 levels of IWT 5/3 or DWT 9/7)	✗	Wavelet transform (3 levels of 9/7 Integer DWT or 9/7 Float DWT)	✗	Discrete cosine transform (DCT) and discrete sine transform (DST)
Prediction	Intra: using 3 neighbor samples	✗	Inter: using 2 channels for spectral prediction	✗	• Intra: using 1 or 4 neighbor samples • Inter: up to 15 channels for spectral prediction	• Intra: using adjacent blocks as reference, 33 directional plus 2 special modes supported. • Inter: up to 15 frames
Quanti- zation	Uniform scalar quantization	• Uniform scalar deadzone quantization (Part-1 of standard) • Variable scalar deadzone quantization, and Trellis coded quantization (Part-2 of standard)	Uniform scalar quantization	Uniform scalar quantization	✗	Uniform scalar quantization
Bitplane coding	✗	Each bitplane is encoded with three coding passes: (1) significance propagation pass, (2) magnitude refinement pass, and (3) clean-up pass. For the first bitplane only clean-up pass is used	✗	First, the first bits of the quantized DC coefficients are encoded. Then, the remaining DC coefficients bit planes are encoded along with the bit planes of AC coefficients using several refinement passes	✗	✗
Entropy coder	Golomb Coder and Run Length Coder	MQ Arithmetic Coder. Contextual binary arithmetic coder. Contexts are defined using the 8 adjacent neighbors	Contextual Arithmetic Coder using up to 1024 contexts	Variable Length Coder and Fixed Length Coder	Golomb Coder	Arithmetic Coder (CABAC with 154 contexts) and Variable Length Coder (CAVLC)

**Table 4:** For each coding technique, the configuration used in the experiments is reported. Default option is employed for the parameters not specified in the table.

Coding Technique	Paradigm	Setting and Mode	Spatial Transform	Spectral Transform
JPEG-LS	Lossless	Plane-interleaved mode	—	• Multilevel Clustering RKLT (200 clusters in first level and multilevel mode) • IWT 5/3 (5 levels) • RPOT • Maximum RWA (Exogenous variant)
	Near-lossless	Plane-interleaved mode	—	—
JPEG 2000	Lossless	Code-blocks of 64×64 size and 1 quality layer	IWT 5/3 (5 levels)	• Multilevel Clustering RKLT (200 clusters in first level and multilevel mode) • IWT 5/3 (5 levels) • RPOT • Maximum RWA (Exogenous variant)
	Lossy	Code-blocks of 64×64 size and 1 quality layer	DWT 9/7 (5 levels)	• Multilevel Clustering KLT (200 clusters in first level and multilevel mode) • DWT 9/7 (5 levels) • POT
M-CALIC	Lossless	Default	—	• Multilevel Clustering RKLT (200 clusters in first level and multilevel mode) • IWT 5/3 (5 levels) • RPOT • Maximum RWA (Exogenous variant)
	Near-lossless	Default	—	—
CCSDS-122.0	Lossless	Default	Default	• Multilevel Clustering RKLT (200 clusters in first level and multilevel mode) • IWT 5/3 (5 levels) • RPOT • Maximum RWA (Exogenous variant)
	Lossy	Default	Default	• Multilevel Clustering KLT (200 clusters in first level and multilevel mode) • DWT 9/7 (5 levels) • POT
CCSDS-123.0	Lossless	Default	—	• Multilevel Clustering RKLT (200 clusters in first level and multilevel mode) • IWT 5/3 (5 levels) • RPOT • Maximum RWA (Exogenous variant)
HEVC	Lossless	Intra and inter prediction	Default	• Multilevel Clustering RKLT (200 clusters in first level and multilevel mode) • IWT 5/3 (5 levels) • RPOT • Maximum RWA (Exogenous variant)

**Table 5:** Computational cost in FLOPs for IWT, RPOT, RWA Maximum, RWA Exogenous, RKLT, and Multilevel Clustering RKLT. *z* is the number of spectral channels, *m* is the number of spatial samples per channel, *y* is the number of rows, *l* is the number of wavelet decomposition levels, *k* is the number of detail channels employed in the prediction level *i* [38], *s* is the number of spectral channels per cluster (s≪z), and *C* is the total number of clusters.

Transform	FLOPs
IWT	$2\times 14(1-\frac{1}{2^{l}})mz$
RPOT	16mz+26zy−12m−28y+11mz+5zy−10m−5y
RWA Maximum	$\begin{array}{c} 8\left(1-\frac{1}{2^{l}}\right)mz+\left(\sum_{i=1}^{l}\left(2m-1\right){\left(k_{i}+1\right)}^{2}+{\left(k_{i}+1\right)}^{3}+\left(\frac{z}{2^{i}}\right)\left(k_{i}+1\right)\left[\left(2m-1\right)+\left(2k_{i}+1\right)\right]\right)+\\ +\left(2\sum_{i=1}^{l}\left(2k_{i}-1\right)m\frac{z}{2^{i}}\right)+2m\left(z-1\right) \end{array}$
RWA Exogenous	$8\left(1-\frac{1}{2^{l}}\right)mz+\left(2\sum_{i=1}^{l}\left(2k_{i}-1\right)m\frac{z}{2^{i}}\right)+2m\left(z-1\right)$
RKLT	$m\left(4z^{2}+3z+1\right)+\frac{32}{3}z^{3}+\frac{1}{2}z^{2}-\frac{37}{6}z+5+m\left(3z^{2}+z-3\right)$
Multilevel Clustering RKLT	$\sum_{c\in C}m\left(4s^{2}+3s+1\right)+\frac{32}{3}s^{3}+\frac{1}{2}s^{2}-\frac{37}{6}s+5+m\left(3s^{2}+s-3\right)$

**Table 6:** Computational cost (in FLOPs) and transform performance (entropy) for different cluster sizes of Multilevel Clustering RKLT. Transform performance results are not provided when $2^{0}$, $2^{1}$, $2^{2}$, and $2^{3}$ clusters are defined in the first level. For these cases, applying the spectral transform would require several days due to the high computational cost, which results impractical in a real scenario.

Number of Clusters Defined in the First Level	Cluster Size	Total Number of Clusters	FLOPs	Entropy
$2^{0}$	$2^{13}$	1	$4.90\times 10^{13}$	-
$2^{1}$	$2^{12}$	3	$3.45\times 10^{13}$	-
$2^{2}$	$2^{11}$	7	$1.95\times 10^{13}$	-
$2^{3}$	$2^{10}$	15	$1.03\times 10^{13}$	-
$2^{4}$	$2^{9}$	31	$5.27\times 10^{12}$	5.20
$2^{5}$	$2^{8}$	63	$2.67\times 10^{12}$	5.14
$2^{6}$	$2^{7}$	127	$1.35\times 10^{12}$	5.14
$2^{7}$	$2^{6}$	255	$6.78\times 10^{11}$	5.13
$2^{8}$	$2^{5}$	511	$3.42\times 10^{11}$	5.13
$2^{9}$	$2^{4}$	1023	$1.74\times 10^{11}$	5.17
$2^{10}$	$2^{3}$	2047	$8.98\times 10^{10}$	5.25
$2^{11}$	$2^{2}$	4095	$4.74\times 10^{10}$	5.48
$2^{12}$	$2^{1}$	8191	$2.56\times 10^{10}$	5.94

**Table 7:** IASI L1C products used in the experiments. Sizes and averaged zero-order entropies per instrument are provided (48 orbits per instrument). *M* is the number of spectral channels, *Ns* is the number of scan lines, *N-FORs* is the number of FORs per line, and *N-IFOVs* is the number of IFOVs per FOR.

Instrument	Size (M × Ns × N-FORs × N-IFOVs)	Average Entropy
IASI-A Products	8461 × (630-787) × 30 × 4	12.84
IASI-B Products	8461 × (742-788) × 30 × 4	12.83
Average	8461 × (761) × 30 × 4	12.83

**Table 8:** Lossless compression of IASI L1C products. Results are reported in compression ratio (higher is better). Percent savings (higher is better) with respect to original technique are provided within brackets.

**IASI-A—Lossless Compression Ratio & Percent Savings**						
	**Tra.**	**No Transform**	**IWT**	**RPOT**	**RWA**	**Multilevel Clustering RKLT**
**Tech**						
JPEG-LS		1.78:1	2.26:1 (21.24%)	2.26:1 (21.24%)	2.44:1 (27.05%)	2.46:1 (27.64%)
JPEG 2000		1.73:1	2.24:1 (22.77%)	2.24:1 (22.77%)	2.43:1 (28.81%)	2.47:1 (29.96%)
M-CALIC		2.32:1	2.32:1 (0.00%)	2.34:1 (0.85%)	2.48:1 (6.45%)	2.54:1 (8.66%)
CCSDS-122.0		1.68:1	2.13:1 (21.13%)	2.13:1 (21.13%)	2.29:1 (26.64%)	2.33:1 (27.90%)
CCSDS-123.0		2.42:1	2.42:1 (0.00%)	2.39:1 (−1.24%)	2.46:1 (1.63%)	2.47:1 (2.02%)
HEVC		2.23:1	2.29:1 (2.62%)	2.28:1 (2.19%)	2.45:1 (8.98)	2.50:1 (10.80%)
**IASI-B—Lossless Compression Ratio & Percent Savings**						
	**Tra.**	**No Transform**	**IWT**	**RPOT**	**RWA**	**Multilevel Clustering RKLT**
**Tech**						
JPEG-LS		1.79:1	2.28:1 (21.49%)	2.27:1 (21.15%)	2.45:1 (26.94%)	2.48:1 (27.82%)
JPEG 2000		1.74:1	2.25:1 (22.67%)	2.25:1 (22.67%)	2.44:1 (28.69%)	2.49:1 (30.12%)
M-CALIC		2.34:1	2.33:1 (−0.43%)	2.35:1 (0.43%)	2.50:1 (6.40%)	2.56:1 (8.59%)
CCSDS-122.0		1.69:1	2.14:1 (21.03%)	2.14:1 (21.03%)	2.30:1 (26.52%)	2.34:1 (27.78%)
CCSDS-123.0		2.44:1	2.44:1 (0.00%)	2.40:1 (−1.64%)	2.48:1 (1.61%)	2.48:1 (1.61%)
HEVC		2.24:1	2.30:1 (2.61%)	2.29:1 (2.18%)	2.47:1 (9.31%)	2.52:1 (11.11%)

**Table 9:** Near-lossless compression of IASI L1C products. Results are reported in compression ratio (higher is better). Results for lossless compression (PAE = 0) are included. Percent savings (higher is better) with respect to lossless compression are provided within brackets.

	IASI-A		IASI-B	
PAE	JPEG-LS	M-CALIC	JPEG-LS	M-CALIC
0	1.78	2.32	1.79	2.34
1	2.17 (17.97%)	3.02 (23.18%)	2.18 (17.89%)	3.05 (23.28%)
3	2.60 (31.54%)	3.90 (40.51%)	2.61 (31.42%)	3.95 (40.76%)
7	3.15 (43.49%)	5.21 (55.47%)	3.18 (43.71%)	5.28 (55.68%)
15	3.93 (54.71%)	7.34 (68.39%)	3.98 (55.03%)	7.48 (68.72%)
31	5.11 (65.17%)	11.11 (79.18%)	5.18 (65.44%)	11.35 (79.38%)
63	6.99 (74.54%)	18.39 (87.38%)	7.08 (74.72%)	18.82 (87.57%)
127	10.00 (82.20%)	33.33 (93.03%)	10.19 (82.43%)	34.04 (93.13%)
255	15.09 (88.20%)	61.54 (96.23%)	15.38 (88.36%)	64.00 (96.34%)

**Table 10:** Compression and decompression runtimes for the coding schemes that produce the best performance for lossless, near-lossless, and lossy compression. The PAE employed for near-lossless compression is 1. The target bit-rate used for lossy compression is 2 bpppc. All times are expressed in minutes.

Runtimes (in Minutes)	Lossless	Near-Lossless	Lossy
Compression	81.7	15	13.4
Decompression	41.4	11.3	6.2

**Table 11:** Compression setting for PCC, M-CALIC, and Multilevel Clustering + JPEG 2000 comparison.

		PCC	M-CALIC	Multilevel Clustering KLT + JPEG 2000
	Compression ratio	PC scores	PAE	Target bit-rate
Experiment 1	9:1	200	19	1.78
Experiment 2	12:1	150	29	1.33
